# Development of a 50K SNP array for whole-genome analysis and its application in the genetic localization of eggplant (*Solanum melongena* L.) fruit shape

**DOI:** 10.3389/fpls.2024.1492242

**Published:** 2024-11-25

**Authors:** Chuying Yu, Qihong Yang, Weiliu Li, Yaqin Jiang, Guiyun Gan, Liangyu Cai, Xinchun Li, Zhiqiang Li, Wenjia Li, Min Zou, Yang Yang, Yikui Wang

**Affiliations:** ^1^ Vegetable Research Institute, Guangxi Academy of Agricultural Sciences, Nanning, China; ^2^ Vegetable and Flower Research Institute, Chongqing Academy of Agricultural Sciences, Chongqing, China

**Keywords:** eggplant, liquid-phase probes, fruit shape, BSA-seq, candidate gene

## Abstract

**Introduction:**

Current eggplant variety breeding is still mainly based on conventional methods, and there remains a lack of effective molecular breeding systems for complex traits controlled by multiple genes, such as yield and quality. To accelerate the research progress of eggplant genetics and molecular breeding, it is necessary to implement a genome-based breeding strategy.

**Methods:**

Therefore, in this study, a SNP array containing 50K liquid-phase probes was designed on the basis of the resequencing data of 577 eggplants.

**Results:**

The developed 50K liquid-phase probes were used to perform targeted capture sequencing on 12 eggplant lines, and the efficiency of probe capture exceeded 99.25%. Principal component, phylogenetic, and population structure analyses divided the 577 eggplants into 7 subgroups, and statistical analysis was performed on the fruit shape and color of the materials in the different subgroups. Further analysis of the geographical distribution of 428 Chinese eggplant materials revealed that the geographical regions of different subgroups were similar. The 50K SNP liquid-phase array was used to perform bulked- segregant analysis combined with whole-genome resequencing (BSA-seq) of fruit shape in the F_2_ population, which consisted of 1435 lines constructed with E421 as the maternal parent and 145 as the paternal parent. The BSA-seq data were located in the 78444173−84449348 interval on chromosome 3, with a size of 6 Mb, which was narrowed to 712.6 kb through fine mapping. Further sequence alignment and expression analysis revealed *SmIQD14* as a candidate gene controlling eggplant fruit shape. The 50K SNP liquid-phase array can be widely used in future eggplant molecular breeding research.

## Introduction

Eggplant (*Solanum melongena* L.) is a vegetable crop of the genus *Solanum* in the family Solanaceae. Its fruit is the main production organ and has important agricultural economic value (Knapp et al., 2013). Fruit shape index (FSI) is an important target breeding trait of eggplant, which mainly includes fruit length (FL) and fruit diameter (FD), has important research value (Gaccione et al., 2023). As an important appearance quality, FSI directly affects the commercial value of eggplant. Eggplant fruit size variation is rich and is controlled by complex quantitative traits. It is a very important component of eggplant genetic research (Uddin et al., 2021). Breeding excellent new varieties is one of the most effective methods for increasing yield, and the breeding of excellent varieties is closely related to the continuous innovation of crop breeding methods. Through the study of key technical methods of crop breeding, the yield and quality of crops can be effectively improved, which greatly promotes crop production. The “Green Revolution” based on dwarfing breeding technology is a representative example (Chai et al., 2022; Huang et al., 2021).

The development of high-throughput, low-cost genotyping technology for breeding is becoming increasingly important (Kockum et al., 2023). Next-generation sequencing (NGS) has been implemented for a new generation of genotyping, such as whole-genome resequencing and simplified genome sequencing, allowing the development of tens of thousands of SNP markers across the entire genome (Kamarudin et al., 2023; Pootakham, 2023). Genotyping by target sequencing (GBTS) is called targeted sequence capture or liquid-phase chip, which is a newly developed genotyping technology based on NGS. GBTS combines the advantages of solid-phase chips and local sequencing and has the characteristics of customization, flexibility, high throughput and low cost (Li et al., 2024). It can accurately capture non-highly repetitive regions at any position and any length of the genome, including scattered points, exons, and genomic fragments. It can also simultaneously detect multiple types of genotype variations, such as SNPs, short simple sequence repeats (SSRs), insertion-deletions (InDels), chromosome structural variations (SVs), known/unknown fusion genes and methylation sites (Guo et al., 2021). Liquid-phase chip technology is widely used, and many reports on the evaluation of animal and plant germplasm resources, genetic map construction, gene positioning and cloning, and molecular marker-assisted selection have been published (Du et al., 2019; Ma et al., 2022; Chen et al., 2022). Using chips to evaluate germplasm resources can successfully distinguish different types and sources, analyze the relationships between different materials, and provide a basis for the mining and combination of excellent parents (Rasheed et al., 2017). For complex traits controlled by multiple genes, such as yield, quality and stress resistance, liquid-phase chips can play a greater role in genome-wide association analysis (GWAS) and genome-wide selection (GS) (Rasheed et al., 2017). For example, Cornell_6K_Array_Infinium_Rice, Affymetrix GeneChip Rice 44 K, and Illumina Infinium Rice SNP50 have been widely used in germplasm resource screening, variety authenticity and purity identification, and genetic background analysis of breeding materials (Yu et al., 2014; Thomson et al., 2017; Chen et al., 2014). The Bnapus 50K liquid-phase chip was developed based on the DNA array data of 505 inbred lines, which can be used for rapeseed gene positioning and variety improvement and identification (Xiao et al., 2021). The results of the detection of 2078 soybean materials based on the GenoBaits Soy40K liquid-phase chip supported the good genotyping, population structure analysis, GWAS and molecular breeding performance of this chip (Liu et al., 2022). A gene associated with flowering time and candidate genes associated with fruit core size were identified through genome-wide association studies based on the pear 200K AXIOM PyrSNP array (Li et al., 2019).

Bulked segregant analysis combined with whole-genome resequencing (BSA-seq) has been applied to the rapid location of quantitative trait loci (QTLs) and target gene mining for important traits in cotton, rice, wheat, peanut, chickpea, corn and other crops (Li and Xu, 2022). The peanut materials of YZ9102 (pink seed coat) were hybridized with those of ZH12 (red seed coat) and ZH2 (red seed coat), and two F_2:4_ populations were constructed. Finally, *AhRt2* was found to be related to the red outer seed coat of peanut (Zhang et al., 2022). Through fine mapping of a BC_1_F_2_ population of upland cotton based on BSA-seq combined with F_2:3_ and F_2:4_ populations, *Ghir_D03G012430* and *Ghir_D03G012390* were finally found on chromosome D03, which may play important roles in cell division and differentiation during the formation of the first fruiting branch node (Jia et al., 2023). In rice, by constructing an F_2_ population for linkage mapping, the *qTGW10-3* interval was narrowed to 75.124 kb. Through molecular marker-assisted selection, candidate genes have been used in breeding (Wang et al., 2023). To analyze the genetic mechanism of radish resistance to Fusarium wilt, *ForRs1* was identified as the main QTL. Fine mapping was performed using the F_2:3_ population, and the *ForRs1* locus was narrowed to a 195-kb region (Ezeah et al., 2023). An increasing number of studies have shown that the initial positioning of QTLs obtained by combining phenotypic identification with genotypic data is not sufficiently accurate. Therefore, it is necessary to construct a larger segregating population for screening and to use suitable exchange plants. Ultimately, the QTL can be locked within a very small interval and sometimes even within a region containing only one annotated gene, thereby further determining the target gene related to the target trait and establishing a good foundation for subsequent functional verification and analysis of molecular mechanisms (Li and Xu, 2022).

Conventional breeding methods, such as hybrid breeding and backcross breeding, are important means to improve eggplant traits. However, owing to the lack of consideration of genomic genetic information, the breeding results are blind, and the output of excellent varieties or lines is low (Anand et al., 2023). With the development of sequencing technology, an increasing number of eggplants have undergone genome sequencing. High-quality genomes provide a good foundation for the genetic positioning and gene mining of important eggplant traits, which is helpful for molecular mechanism analysis and functional marker development and accelerates the molecular breeding of target traits (Wei et al., 2020b; Hirakawa et al., 2014; Song et al., 2019). By using this genomic genetic information, breeders can improve eggplant varieties more efficiently and accurately, thus playing a strong role in promoting the development of the eggplant industry and variety improvement. Therefore, it is necessary to develop a genotyping platform with the characteristics of high throughput, high efficiency, high flexibility and low cost to provide a powerful genotyping tool for subsequent genomics and molecular breeding research on eggplants. To achieve this goal, 577 eggplant germplasm resources were collected from around the world, and a 50K (50836 SNP) liquid-phase chip was developed through resequencing and used to perform genotyping on self-selected 12 eggplant lines to verify the detection effect of the chip. A systematic evolutionary analysis of 577 eggplant accessions was conducted through principal component, phylogenetic, and population structure analyses. Moreover, the QTL for fruit shape was located based on an F_2_ population using a chip, and a candidate gene regulating eggplant fruit shape was discovered through further fine mapping combined with sequence comparative analysis. In summary, the 50K liquid-phase chip developed in this study can be widely used in future molecular breeding research, such as eggplant germplasm resource evaluation, genetic map construction, and gene positioning.

## Materials and methods

### Plant materials

A total of 577 eggplant germplasm resources, which were collected and preserved by the eggplant research group of the Institute of Vegetables, Guangxi Academy of Agricultural Sciences, were used as test materials. Of these, 390 were Chinese varieties (including 78 were purified offspring of self-selected varieties, and self-selected 4 eggplant lines), 148 were varieties from other countries (**Supplementary Table S1**). Seeds with good maturity, full grains and uniformity were selected from all the materials. After being soaked in 55°C warm water for 8 hours, they were placed in a 30°C constant-temperature box for germination. After the seeds turned white, they were sown in 72-well seedling trays. The substrate was peat soil, perlite or vermiculite (3:1:1 ratio). The seedlings were raised in the experimental greenhouse built at the base of Guangxi Academy of Agricultural Sciences. The seedling raising environment included a 27-30°C day temperature, 22°C night temperature, full sun, and dry and wet conditions during the seedling stage. When the plants grew to have 6 leaves and 1 heart, they were moved to the research base of Guangxi Academy of Agricultural Sciences (108°06′38″ E, 23°25′25″ N) and planted in double rows with a row spacing of 140 cm and a plant spacing of 65 cm. The fruit shape index (FSI = longitudinal diameter/transverse diameter) was measured after the eggplants were fully expanded. The longitudinal diameter was from the base of the fruit stalk to the top of the fruit, and the transverse diameter was the maximum cross-sectional width of the fruit. The measurement was performed using an industrial electronic vernier caliper with a length of 0.5 m and an accuracy of 0.01 mm. Descriptive statistical analysis of the F_2_ population was performed using the describe function in the Hmisc package of R language.

### Genotype data collection and processing

**Figure 1:**
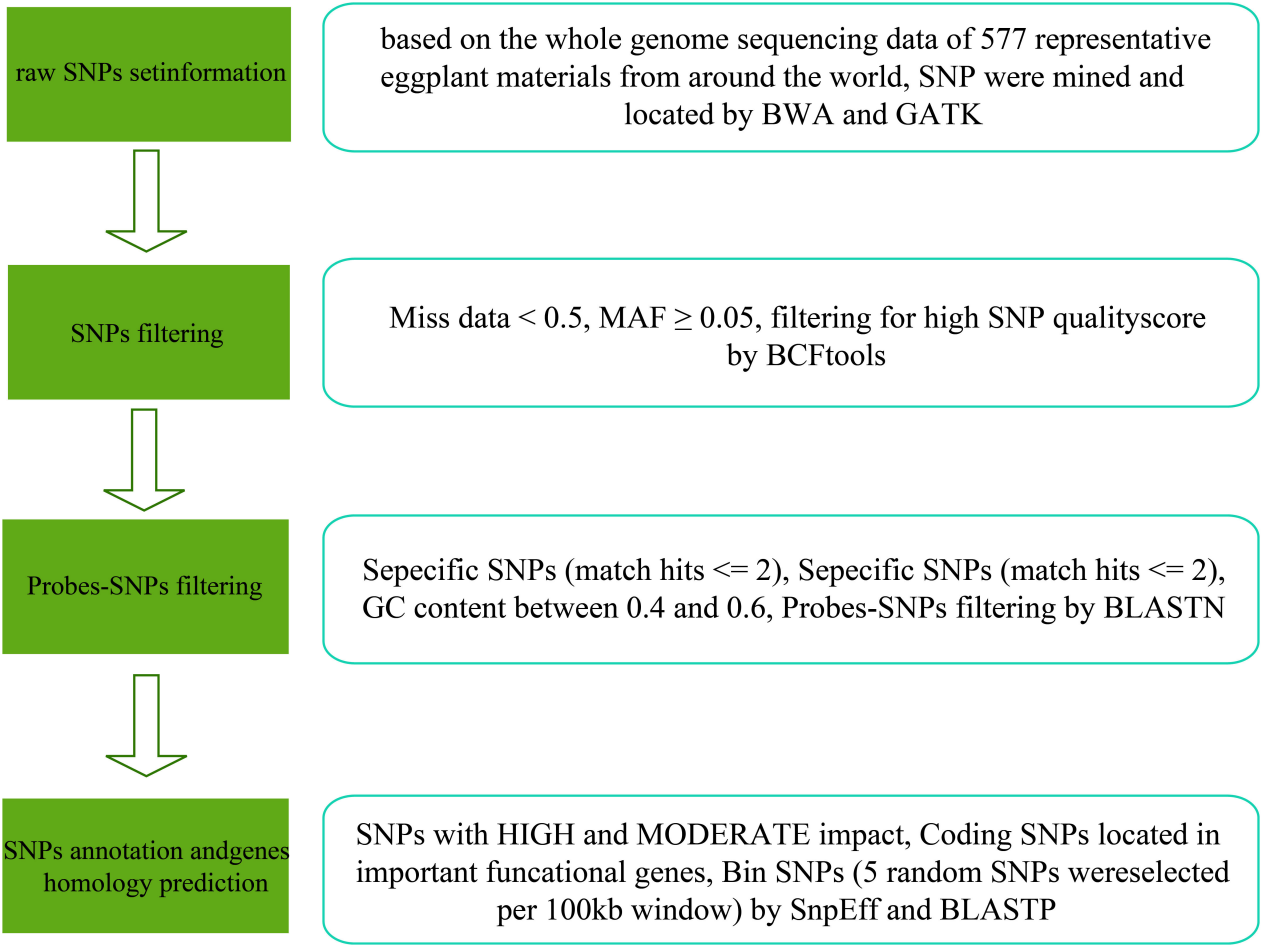
The SNP selection process for the 50K liquid-phase array of eggplant.

A total of 577 young eggplant leaves were collected and stored at -80°C. The genomic DNA was extracted from each sample using the CTAB method. After the sample genomic DNA test was qualified, the DNA was fragmented via mechanical shearing (ultrasound). Subsequently, the fragmented DNA was purified and end-repaired, a 3′-end A was added, and sequencing adapters were connected (Meyer and Kircher, 2010). PCR amplification was performed to form a sequencing library. The constructed library was first subjected to library quality inspection, and the qualified library was sequenced using the Illumina platform. The raw data were filtered using fastp software, which included the adapter sequence, reads with N ratios greater than 5%, and low-quality reads to obtain clean data (Chen et al., 2018). The clean data were aligned to the reference genome of eggplant GUIQIE-1 using BWA software, and SNP variation detection was performed using GATK (Li et al., 2021; Li and Durbin, 2009; McKenna et al., 2010). After further filtering, SNPs for the synthetic chips were obtained (**Figure 1**).

### Population genetics analysis

By obtaining the SNP sites of the chip, MEGA X software, which is based on neighbor-joining (NJ) and the Kimura 2-parameter model, was used for bootstrapping, which was repeated 1,000 times, and a phylogenetic tree was constructed (Kumar et al., 2018). With respect to SNPs, admixture software was used to analyze the population structure of the research material (Montana and Hoggart, 2007). For the research population, the number of subgroups (K value) was preset to 2-10 for clustering, and the clustering results were cross-validated. The optimal number of clusters was determined based on the lowest value of the cross-validation error rate. The R language Pophelper package was used to generate a Q matrix stacking diagram for each K value (Francis, 2017). The smartpcr program in the EIGENSOFT software package was used to perform principal component analysis on the SNP data to obtain the clustering of the samples (Gurinovich et al., 2019).

### Liquid-phase hybridization probe capture

After genomic DNA fragmentation (200-300 bp), generation of the DNA fragment end-repair connection adapter Pre-PCR amplification library, probe hybridization with the target region, and streptavidin affinity-labeled magnetic bead capture using the hybridization probe, the enriched target fragments were eluted and captured Post-PCR amplification. Through the Post-PCR amplification method, the target region is enriched without sacrificing amplification efficiency and specificity, and the target region site is integrated into the amplicon to improve the sensitivity of detection. The captured target region was then sequenced using second-generation sequencing. Fastp software was used to filter the raw data to obtain clean reads, and BWA software was used to compare the clean reads with the reference genome (Chen et al., 2018; Li and Durbin, 2009). The clean reads were located on the reference genome through comparison, and the sequencing depth, genome coverage and other information for each sample were counted, after which mutation detection was performed.

### BSA-seq and fine mapping

F_1_ was obtained by hybridization of E421 (maternal parent) and 145 (paternal parent). The F_2_ population containing 1435 lines was obtained via self-pollination of F_1_ plants. From these lines, 30 lines with extreme phenotypes (i.e., plants with fruit shape indices ≤0.9 and >1.2) were selected, and the total DNA was extracted and mixed in equal amounts to construct round and oval fruit pools. The genotypes were determined using a chip, and the candidate regions of the target genes were analyzed using the Euclidean distance (ED) method (Takagi et al., 2013). The SNP sites with different genotypes between the two pools were used to count the depths of each base in different pools, and the square of the original ED was taken as the correlation value to eliminate background noise. The SNPNUM method was subsequently used to fit the ED value (Hill et al., 2013). Based on the initial positioning results of BSA-seq, 6 pairs of markers with polymorphic differences between parents were uniformly designed in this interval. The 1435 F_2_ plants were genotyped, and the exchange plants were selected for further fine mapping.

### qRT-PCR

Total RNA was extracted using the RNAprep Pure Polysaccharide and Polyphenol Plant Total RNA Extraction Kit (Tiangen, China). The concentration of each RNA sample was determined using a NanoDrop 2000 spectrophotometer (Thermo Fisher Scientific, Waltham, MA, USA). The RNA was reverse transcribed using the M-MLV RTase cDNA Synthesis Kit (TaKaRa, Japan) to obtain cDNA. Real-time PCR amplification was performed on a Bio-Rad CFX96 Real-time System. The iTaq Universal SYBR Green Supermix (Bio-Rad, USA) kit was used according to the provided method, and the amplification system had a volume of 20 μL. The reaction procedure was 95°C predenaturation for 30 s, 95°C denaturation for 5 s, and 60°C annealing for 30 s for 40 cycles. The results were analyzed by relative quantification using the 2^-ΔΔCt^ method (Livak and Schmittgen, 2001). The internal reference gene was actin, and three biological replicates were used for each procedure. All primers used in this study are shown in **Supplementary Table S2**.

## Results

### Resequencing of 577 natural eggplant populations and development of a 50K SNP liquid-phase array

**Figure 2:**
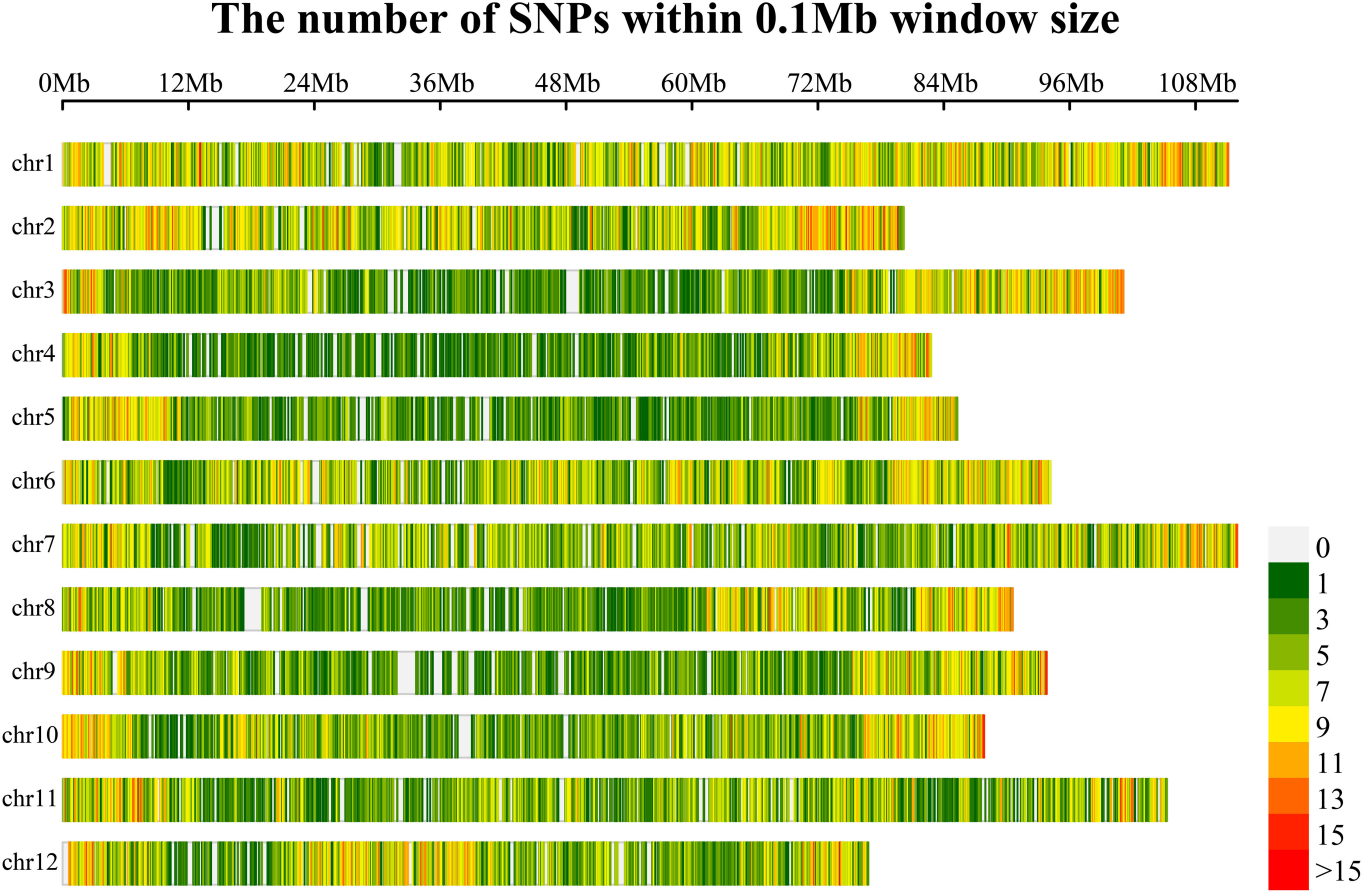
Chromosome distribution density of SNPs in the eggplant 50K liquid-phase array.

In this study, a total of 6662.5 Gb of clean data were obtained after filtering through the resequencing data of 577 eggplant samples. The sequencing depth of the samples was 5.37X~35.86X, the Q20 was between 94.24%~98.43%, the Q30 was between 83.97%~94.84%, the GC content was distributed between 36.10%~40.17%, and the effective number of reads compared with the reference genome was 86.90~99.90%, resulting in 42686847 SNPs (**Supplementary Table S3**). According to the minor allele frequency (MAF: 0.05) and a missing rate of less than 50%, highly consistent SNP sites were obtained. The 100-bp sequence upstream and downstream of the marker (SNP) was extracted, and the whole genome sequence was blasted. The alignment length was greater than 60% (120 bp) and was considered to be a copy. Only single-copy sites were retained. The GC content 100 bp upstream and downstream of the SNP site was greater than 40% and less than 60%, respectively. There was no N sequence within 100 bp upstream or downstream of the SNP site. The locations of the SNPs included mainly intergenic regions, gene coding regions (nonsynonymous mutations, start codons, stop codons, etc., that cause gene functional variations and nonfunctional variations such as synonymous mutations) and gene noncoding regions. Finally, 50,836 SNPs were obtained for the synthesis of the eggplant 50K liquid-phase chip. The distribution of 50,836 SNPs in the eggplant genome revealed that these SNPs were essentially evenly distributed on the chromosomes, with a slightly greater density of SNPs near the telomere region, which was consistent with the distribution pattern of genes on the chromosomes (**Figure 2**; **Supplementary Table S4**). Based on the eggplant 50K liquid-phase chip, self-selected 12 eggplant lines were captured via liquid-phase hybridization capture technology. After the target region sequence was captured and enriched, high-throughput sequencing was performed using a second-generation sequencing platform. The alignment rate exceeded 99.36%, and the detection rate of SNP sites on the 50K liquid-phase chip exceeded 99.25% (**Supplementary Table S5**). These results demonstrated the good repeatability and accuracy of the eggplant 50K liquid-phase chip we developed.

### Population structure and kinship analysis

**Figure 3:**
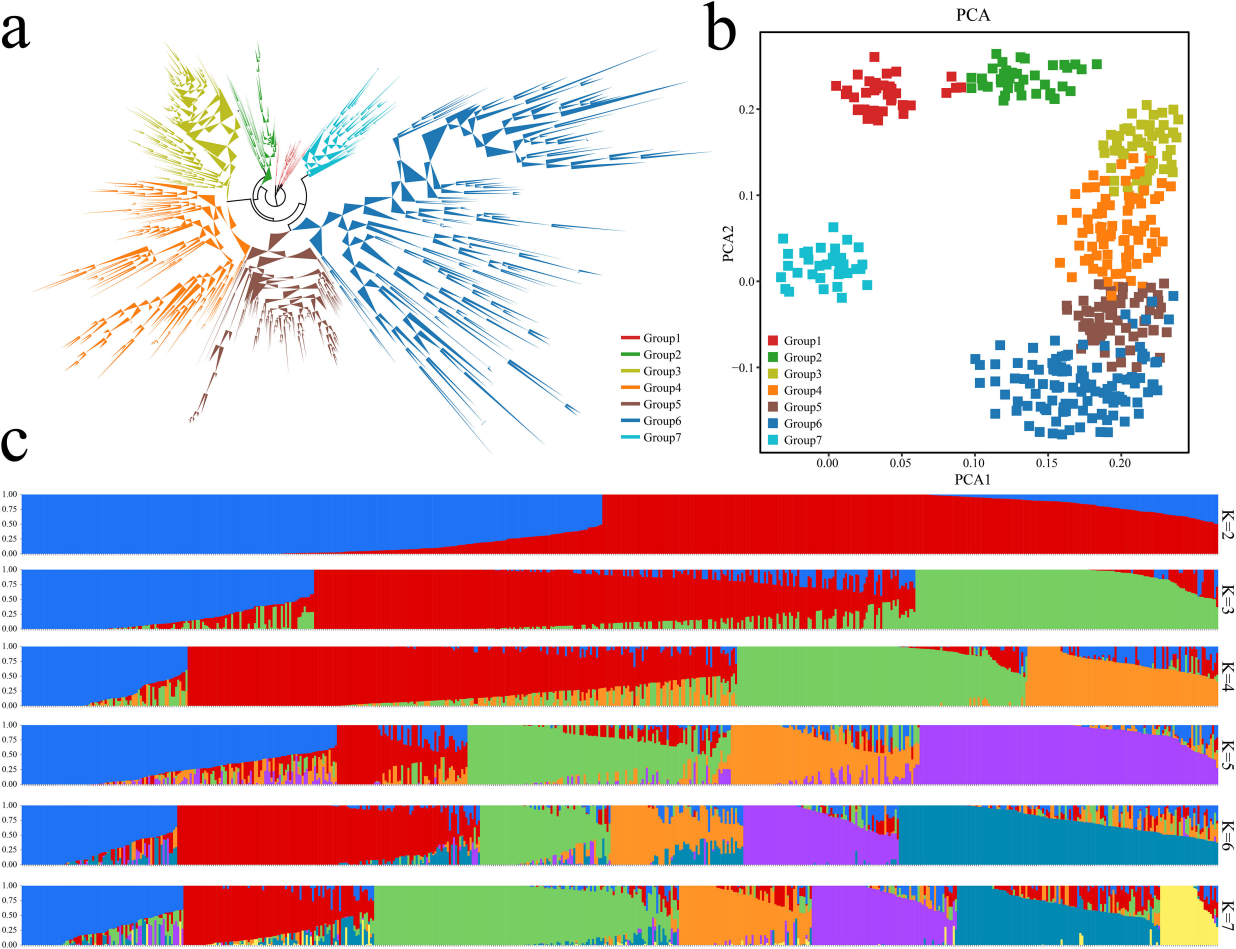
Analysis of population structure and phylogenetic relationships of 577 eggplant samples based on the 50K liquid-phase microarray. **(A)** Analysis of 577 eggplant evolutionary trees; different colors represent different groups. **(B)** PCA of 577 eggplants; different colors represent different groups. **(C)** Analysis of the population structure of 577 eggplant samples; different colors represent different groups.

The phylogenetic analysis of 577 eggplant samples was performed on the basis of the SNP data from the eggplant 50K chip. The evolutionary tree results revealed that all eggplant materials could be divided into 7 groups (**Figure 3A**). Principal component analysis was further performed using the SNPs of the samples, and the grouping information was marked in different colors in the principal component analysis scatter plot. The results of the principal component analysis were consistent with the phylogenetic tree results (**Figure 3B**). PCA distinguished the 577 eggplant materials well, and the eggplant germplasms of Groups 3, 4, 5 and 6 were closely related. The coefficient of variation error (CV error) of the samples under different K values was calculated, which revealed that the coefficient of variation error rate was the lowest when K=7 (**Supplementary Figure S1**). Therefore, it was more appropriate to divide the analyzed eggplant samples into 7 subgroups. All the samples were divided into 7 groups based on the clustering results of the genetic structure data (**Figure 3C**). The average linkage disequilibrium (LD) coefficient (r^2^) at a distance of 40 kb on the genome is about 0.4, but at a distance of 100 kb, the corresponding average LD coefficient is reduced to 0.3. At a distance of more than 200 kb, the corresponding average LD coefficient is more than 0.2. This may be due to the high genetic diversity of the population we selected (**Supplementary Figure S2**).

**Figure 4:**
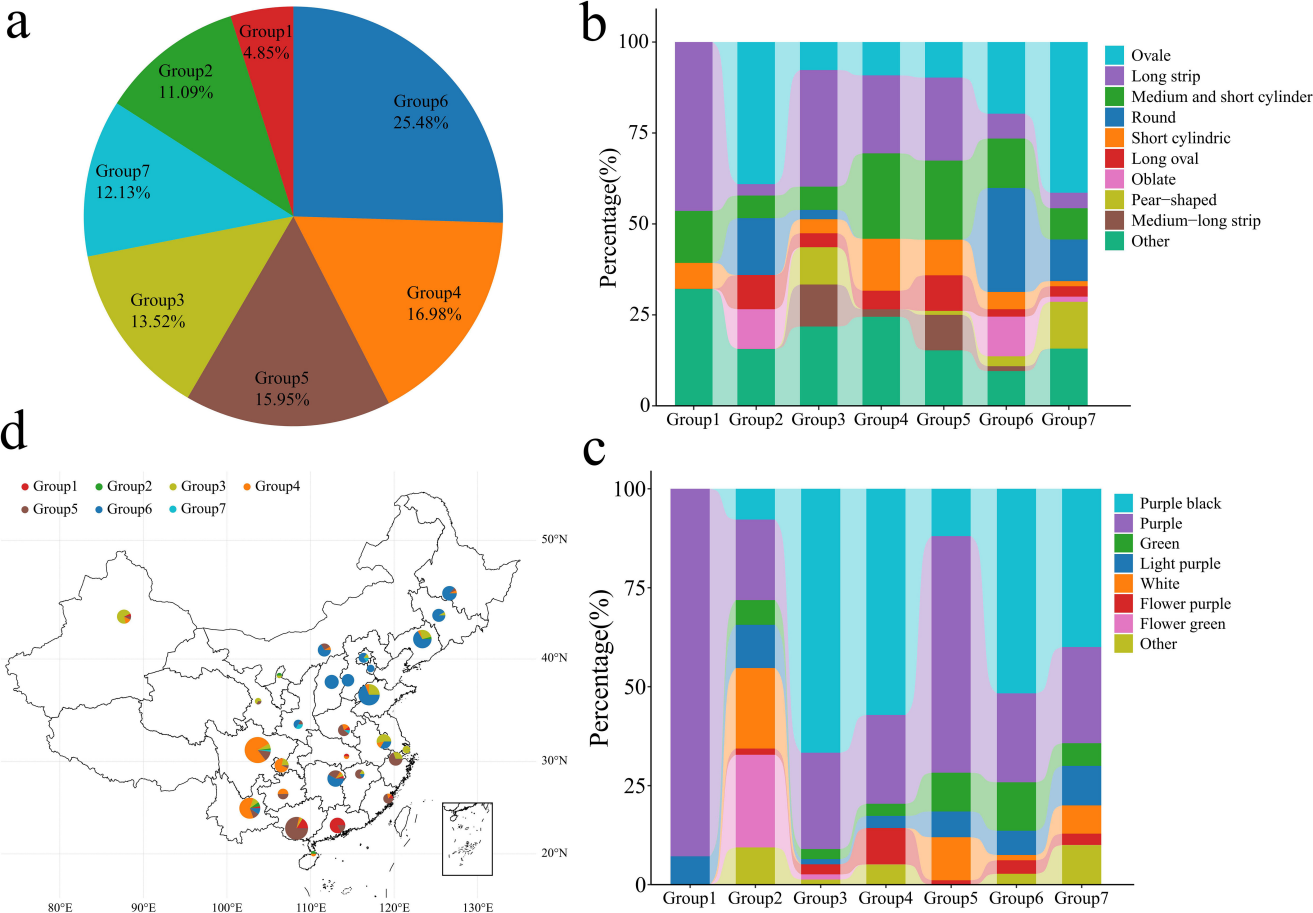
Classification, fruit shape and color analysis of 577 eggplant samples. **(A)** Proportion of 577 eggplant materials; different colors represent different groups. **(B)** Proportion of different eggplant fruit shapes in 577 groups. **(C)** The proportion of fruit colors in different groups of 577 eggplants. **(D)** Geographical distribution of Chinese eggplant materials; different colors represent different groups.

Through the phylogenetic tree of the samples and the PCA classification results, 577 eggplant materials in the 7 groups were statistically analyzed (**Figure 4A**). Group 1 contained 28 genotypes, accounting for 4.85% of the total genotypes. Group 2 contained 64 genotypes, accounting for 11.09% of the total genotypes. Group 3 contained 78 genotypes, accounting for 13.52% of the total genotypes. Group 4 contained 98 genotypes, accounting for 16.98% of the total genotypes. Group 5 contained 92 genotypes, accounting for 15.95% of the total genotypes. Group 6 contained 147 genotypes, accounting for 25.48% of the total genotypes. Group 7 contained 70 genotypes, accounting for 12.13% of the total genotypes. Further analysis of the fruit shape of the 577 eggplants revealed that the fruits in Groups 1, 3, 4 and 5 were mainly long strips, medium‒short tubes or short tubes (**Figure 4B**). The fruits in Groups 2, 6 and 7 were mainly oval, round and flat. Analysis of 577 eggplant fruits revealed that the fruits in Groups 1 and 2 consisted of mainly light purple and purple‒red fruits. The fruits in Groups 3, 4, 5 and 6 were mainly purple‒black and purple‒red (**Figure 4C**). Further analysis of the geographical distribution of the 390 Chinese eggplant materials revealed that Group 1 eggplant materials were distributed mainly in Guangxi and Guangdong, China (**Figure 4D**). Groups 4 and 5 were distributed mainly in southern China (Yunnan, Guangxi, Chongqing, Guangdong, Zhejiang and Fujian). Group 6 was distributed mainly in the central and eastern coastal areas of China (Shandong, Liaoning, Shanxi, Heilongjiang, Hunan, Jilin, Hebei, Inner Mongolia, Jiangsu, Beijing, Tianjin, Yunnan, Shaanxi and Jiangxi). Groups 2 and 7 consisted mainly of materials from other countries, with small amounts of materials from Yunnan, Liaoning, Hainan, Ningxia, Beijing, Sichuan, Anhui and Shaanxi in China. An analysis of the phylogenetic tree, PCA, population structure and geographical distribution revealed that eggplants of the same subpopulation had a close geographical distribution. An analysis of the phylogenetic tree, PCA, population structure and geographical distribution revealed that eggplants of the same subpopulation had a close geographical distribution.

### Liquid-phase chip BSA-seq of eggplant fruit shape

**Figure 5:**
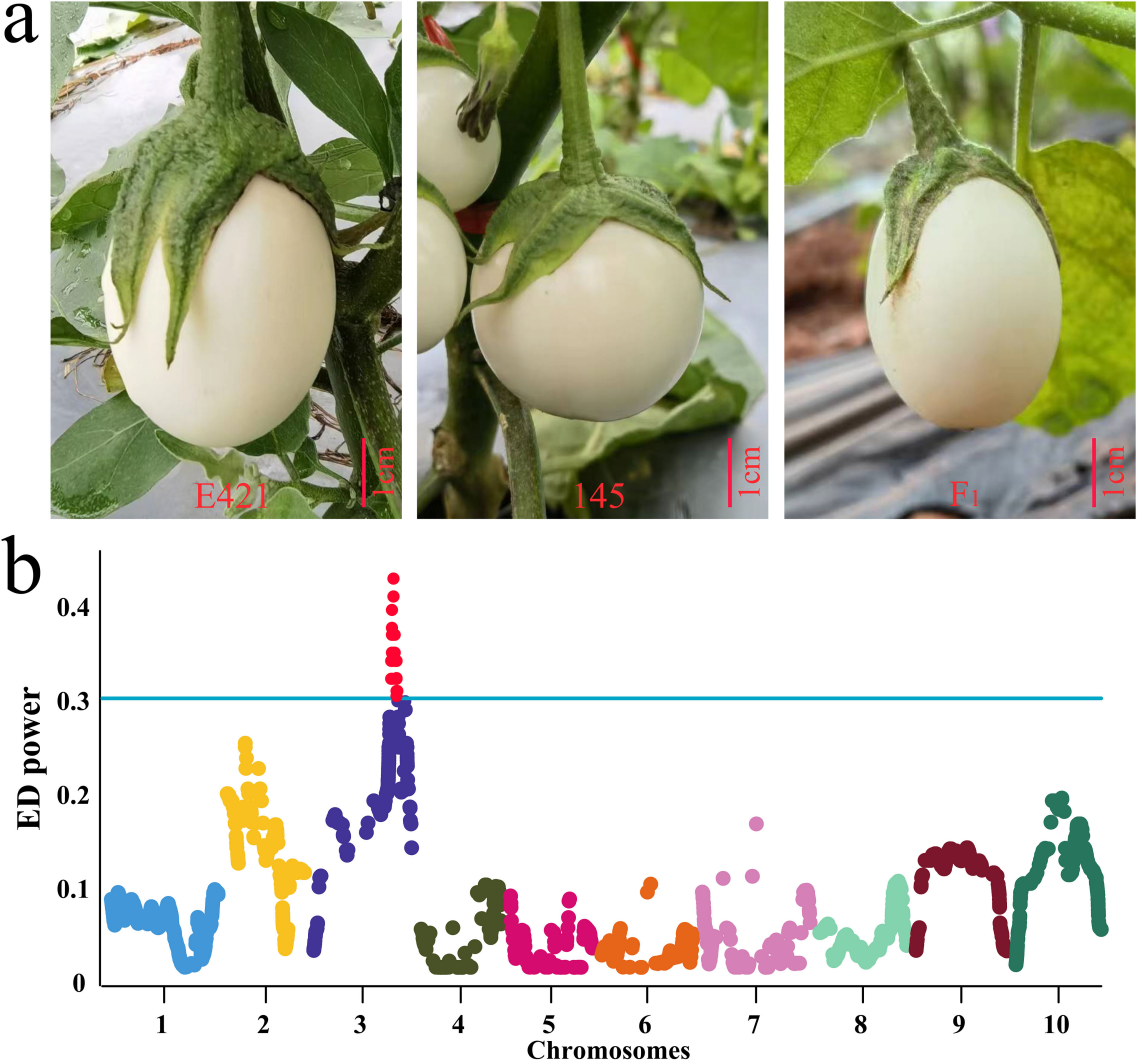
Results of QTL mapping for the phenotype and fruit shape of E421, 145 and F_1_. **(A)** Fruit shape phenotypes of E421, 145 and their hybrid F_1_ progeny, bar = 1cm. **(B)** BSA-seq results for the fruit shape of eggplant E421 and 145 hybrid F_2_ generations using a 50K liquid-phase array.

An F_2_ population of 1435 lines was constructed with E421 (oval fruit, FSI=1.2) as the female parent and 145 (round fruit, FSI=0.9) as the male parent. The F_1_ fruit was oval (The mean of FSI is 1.2 and the standard deviation is 0.04), and the F_2_ fruit was round or oval (**Table 1**; **Figure 5A**). Statistical analysis of the 1435 F_2_ lines revealed that 1084 lines (FSI > 1) had oval fruit and 351 lines (FSI ≤ 1) had round fruit, which conformed to the 3:1 Mendelian inheritance law (χ^2^ = 0.22 < χ^20.05,1^ = 3.84, *P* > 0.05) (**Supplementary Figure S3**). A single dominant gene controlled the oval fruit shape of E421. Thirty lines with FSI ≤ 0.9 and thirty lines with FSI >1.2 were selected for BSA-seq, respectively. The eggplant 50K liquid-phase probe was subsequently used for genotyping. The gene of interest was located in the QTL interval of 78444173-84449348 on chromosome 3 according to the ED algorithm (**Figure 5B**).

**Table 1:** FSI of E421 and 145 and their F_2_ populations in 1435 lines.

Parent				F_2_						
	E421	145	Diff	mean	sd	min	max	skew	kurtosis	CV(%)
FSI	1.2 ± 0.04	0.9 ± 0.01	**	1.08	0.09	0.79	1.3	-0.4	-0.49	8.33

Diff, difference; ** *p* < 0.01; sd, standard deviation; CV, coefficient of variation.

### Fine mapping and candidate gene mining of eggplant fruit shape

**Figure 6:**
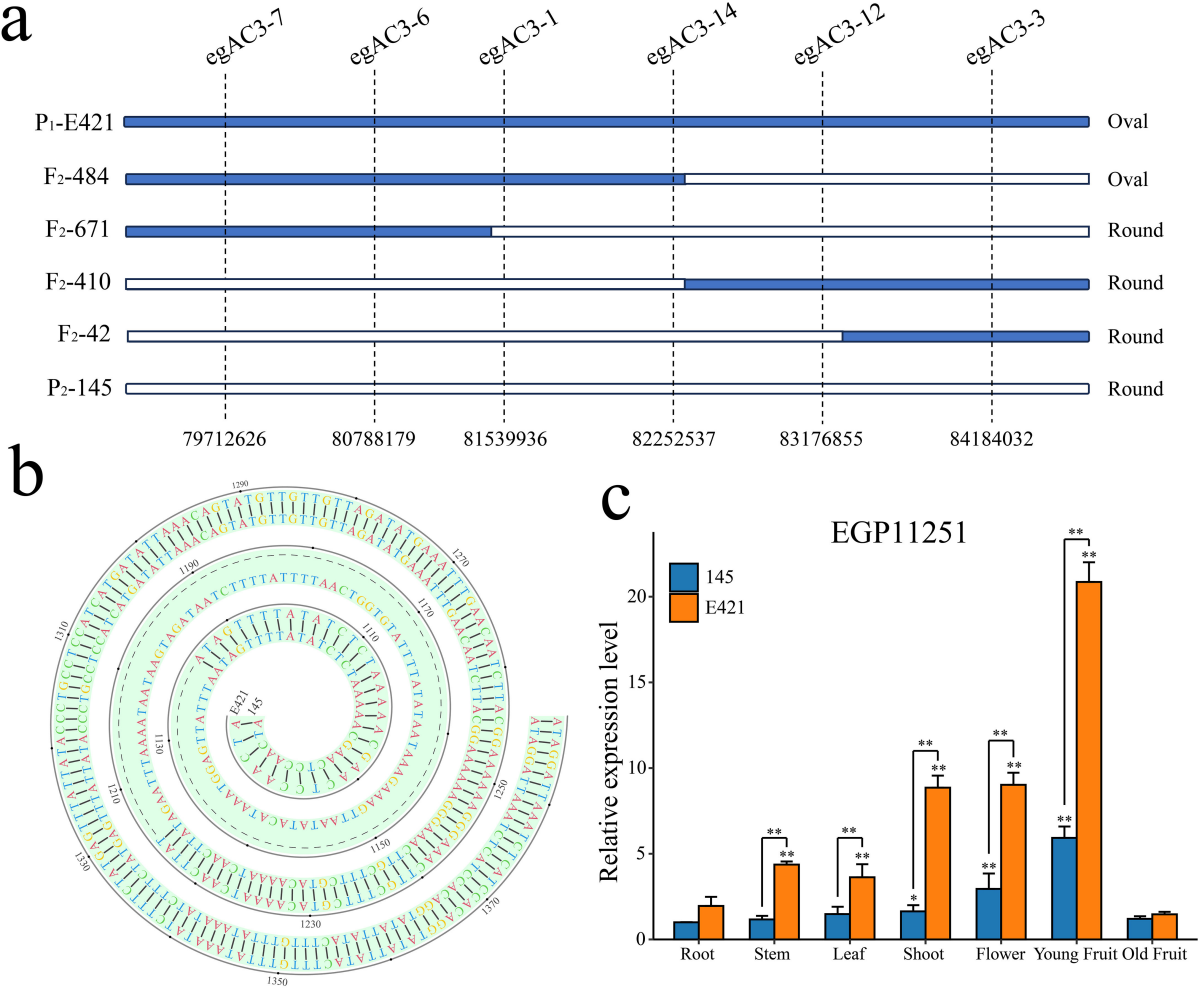
Fine mapping of QTLs for fruit shape and identification of candidate genes in eggplant. **(A)** Fine mapping of chromosome 3 for eggplant fruit shape. **(B)** Comparative analysis of the sequences of *SmIQD14* in the E421 and 145 parents. **(C)** Analysis of the expression pattern of *SmIQD14* in the E421 and 145 parents, n= 3, *p < 0.05, **p < 0.01.

Based on the BSA-seq positioning results, 6 pairs of SNP markers with polymorphisms between parents were evenly designed in the 78444173-84449348 interval on chromosome 3. The above markers were used to genotype 1435 F_2_ lines, and the target gene was located between markers egAC3-1 and egAC3-14 combined with the phenotype. The two wings contained 1 and 2 exchange plants, with physical intervals of 81539936-82252537 and a distance of 712.6 kb (**Figure 6A**). This interval contains a total of 27 coding genes. The full-length sequences of the 27 genes (including CDS and 3kb sequence upstream of the promoter) were amplified using DNA from both parents. Sanger sequencing revealed that *EGP11251* had a 91bp Indel in the promoter region (**Figure 6B**). The CDS and 3kb upstream of the other 26 genes no polymorphic SNP or INDEL sites were found in the parents. *EGP11251* was further analyzed as a candidate gene controlling eggplant fruit shape. Further analysis of the promoter’s cis-acting elements revealed that the 91bp Indel resulted in the lack of a W-box (TTGACC) motif, which has a core sequence of (C/TTGACT/C) and acts as a binding site for WRKY transcription factors. The tetramer sequence TGAC of the W-box element is highly conserved and is essential for the binding of the WRKY transcription factor family. The gene was annotated as the IQD14 protein, which belongs to the IQD protein family, and we named it *SmIQD14*. qRT-PCR analysis of *SmIQD14* expression in different tissues of the two parents revealed that it was highly expressed at the apical growth point, in flowers, and in young fruits and that the expression level of the gene was significantly greater in the oval material E421 than in the round-fruited material 145 (**Figure 6C**).

## Discussion

Conventional breeding based on phenotypic selection is currently the mainstream breeding technology (Alemu et al., 2024). However, owing to the influence of human, financial and material resources on phenotypic identification and screening, the number of identified germplasms is very limited, and the identification results are strongly affected by the environment. The use of molecular marker-assisted selection (MAS) is an effective method for screening target traits (De Mori and Cipriani, 2023). In the past decade, the rapid development of next-generation sequencing (NGS) and the continuous decline in sequencing costs have enabled the rapid development of genomics in recent years. An increasing number of animal and plant genomes have been assembled and published, thereby laying the foundation for identifying markers in the whole genome of crops on the basis of sequencing data. When a small number of molecular markers (less than 100) are needed to identify the genotype of a large population, low-density Kompetitive Allele Specific PCR (KASP) technology is an effective choice, while high-density SNP markers are still indispensable when conducting QTL positioning, GWAS analysis and GS studies (Ige et al., 2022). In theory, whole-genome sequencing can detect most of the variation, but the sequencing cost is still relatively high. SNP markers have become important tools in crop genome research and molecular breeding. Traditional solid-phase breeding chips are limited in the application of molecular marker-assisted selection because of their high price and limited samples (Rasheed et al., 2018). Liquid-phase breeding chips are based on DNA capture and targeted sequencing and have the characteristics of low cost, high repeatability and reliability, and low requirements for platforms and support systems. At present, multiple high-density liquid-phase chips developed using GBTS technology have been widely used in crop variety selection and molecular breeding design (Ma et al., 2022; Du et al., 2019; Chen et al., 2022; Rasheed et al., 2017; Yu et al., 2014). Liquid-phase chips have been developed for crops such as wheat, rice, corn and soybean, and multiple QTL loci related to key traits have been mined, which can be used to effectively detect key variant types of functional genes (Thomson et al., 2017; Chen et al., 2014; Xiao et al., 2021; Liu et al., 2022; Li et al., 2019). The development of these chips has greatly improved plant germplasm resource evaluation, DNA fingerprint identification, and molecular marker-assisted breeding, among other endeavors. However, an increasing number of studies have shown that the use of a wide variety of resource materials is conducive to mining rare alleles that can be found in only a small number of samples (Kumar et al., 2010). In this study, based on the resequencing data from 577 eggplant germplasm resources, a set of 50K (50,836 SNPs) high-density liquid-phase chips was developed for eggplant using GBTS technology for the first time. Twelve eggplants were typed using 50K liquid-phase chip markers, with a matching rate of more than 99.36% and a SNP site detection rate of more than 99.25%, further verifying the reliability of the chip. These variations represent representative whole-genome variations in eggplant, providing valuable resources for the biology and breeding research on eggplant.

The eggplant has rich intraspecific variation, significant differences between different varieties, and large variations in fruit morphology. Bauhin was the first scholar to classify eggplant into two types, namely, thornless and thorny, on the basis of whether the fruit has thorns (Bauhin, 1980). Bailey (1929) divided eggplant cultivars into three varieties on the basis of fruit shape and plant morphological characteristics, namely, round, long and clustered eggplants. Hara (1944) added four categories to Bailey’s classification, American round, green, snake and thousand-year-old eggplants, reaching a total of seven categories. The research team led by Lester at the University of Birmingham divided eggplant and its related species into eight subgroups based on morphological, seed coat scanning, hybridization experiment, seed protein electrophoresis, allozyme and DNA data (Lester and Hasan, 1991). China has a long history of cultivating eggplant, with a wide variety of eggplant types and varieties, and is considered to be the secondary origin of this vegetable. We used the SNP data from the eggplant 50K chip to conduct a phylogenetic analysis of 577 eggplant samples. The results of the evolutionary tree, PCA and population structure analyses revealed that all eggplant materials could be divided into seven groups, potentially because the materials we selected lacked the wild-type eggplant branch, resulting in 7 subgroups. Groups 4, 5 and 6 can distinguish materials from southern and northern China. Groups 2 and 7 consisted mainly of materials from other countries, whereas China contained a small amount of materials. According to the traditional preferences for eggplant, China could be divided into six regions, namely, the three northeastern provinces and the eastern part of Inner Mongolia, where long stick-shaped eggplants were preferred; the Yangtze River Delta and Taiwan Province, where long strips of eggplant were eaten; the southwestern region, which utilized long and short fruit shapes and stick-shaped and oval eggplants; the northwestern region, which was accustomed to oval or high round eggplants; and Beijing, Tianjin, Henan, Hebei, Shandong and Shanxi, where all round eggplants were accepted. This finding was essentially consistent with the results of our phylogenetic analysis, which also showed that the kinship of eggplants was affected mainly by the geographical origin and that the genetic distance of materials from similar sources was relatively close. This phenomenon might be explained by the similar use of materials by people in the same region, such as eggplant types and breeding units, resulting in eggplant varieties or strains from some of the same regions tending to gather together.

The fruit of wild eggplant is relatively short, usually only 4-5 cm, and it is oblate or spherical (Balkaya, 2017). Fruit size and shape are very important traits in the process of eggplant domestication and improvement (Barchi et al., 2021). The QTL positioning of eggplant fruit shape by genetic means and the identification of candidate genes by marker screening and verification constitute the genetic basis for eggplant fruit shape MAS and constitute important means to analyze the molecular mechanism and core regulatory network of eggplant fruit shape. Eggplant fruit shape is a quantitative trait that conforms to the major gene-polygene inheritance model. At present, some progress has been made in the identification of eggplant fruit shape QTLs (Mangino et al., 2021). Wei et al. detected 4 fruit shape index-related QTLs located on chromosomes 1 and 3, with contribution rates of 6.92%, 8.71%, 10.92%, and 10.6%, respectively (Wei et al., 2020a). Composite interval mapping was performed using the genotype data of 736 molecular markers from an interspecific hybrid population of cultivated eggplant and its wild relative, and a QTL associated with the fruit shape index was located on chromosome 3 (Frary et al., 2014). We also located the QTL of the eggplant fruit shape index on chromosome 3 via BSA-seq using eggplant 50K chip, which indicated that the eggplant fruit shape index was regulated by a major gene on chromosome 3. This is consistent with the positioning results of Wei et al. on fruit shape, further fine mapping narrowed this interval to 712.6 kb (Wei et al., 2020a). Most studies have shown that BSA-seq has no obvious defects in locating QTLs. Despite flaws, molecular marker typing of extreme progeny rather than screening in hundreds of materials is relatively easier to implement in breeding plans and may be converted into meaningful markers (Martina et al., 2024). The ultimate goal of QTL positioning is to mine major effect genes and develop molecular markers to facilitate production practice (Wang et al., 2024). MAS is undoubtedly one of the most effective breeding technologies at present, with important significance and broad application prospects (Joshi et al., 2019). The markers egAC3-1 and egAC3-14 screened by fine mapping in this study can be used as important markers for later eggplant MAS breeding, which can help breeders obtain targeted breeding materials more conveniently and accurately and accelerate the process of determining the eggplant fruit shape.

At present, the candidate genes for fruit shape that have been cloned and studied include mainly the fruit elongation gene SUN, which belongs to the plant-specific IQ domain (IQD) family and affects the shape of plant fruits (Zhang et al., 2024). There have been many reports on the cloning of SUN genes and the regulatory mechanism of fruit or seed shape in crops such as tomatoes, watermelons, cucumbers, melons and rice (Wang et al., 2019; Yang et al., 2013). For example, overexpression of the *CsSUN* gene in cucumber causes the ovary to enlarge. The reason for this phenomenon is not that *CsSUN* promotes cell division but rather that when the fruit is in the ovary stage, *CsSUN* inhibits cell expansion, resulting in a greater number of cells than those in the control, which ultimately leads to fruit elongation (Wang et al., 2019). *OsIQD14* is highly expressed in rice seed coat cells and is induced by auxin. When the rice *OsIQD14* gene is missing, the rice grains are wide and short, and the weight increases. When *OsIQD14* is overexpressed, rice grains are slenderer, but their weight is not strongly affected (Yang et al., 2020). Through fine mapping combined with sequence comparison analysis, we found a 91-bp deletion difference in the promoter region of *SmIQD14* between E421 (oval) and 145 (round fruit). *SmIQD14* is homologous to *SUN*, the main gene regulating long round fruit in tomato (Xiao et al., 2008). Expression analysis revealed that *SmIQD14* was highly expressed in the top growth point, flowers and young fruits, and the expression level of this gene was significantly greater in the oval material E421 than in the round fruit material 145. These findings indicated that *SmIQD14* we found is the same type of gene as SUN, and whether their regulatory mechanisms are the same requires further verification.

## Data Availability

The datasets presented in this study can be found in online repositories. The names of the repository/repositories and accession number(s) can be found in the article/**Supplementary Material**.

## Supplementary material

Supplementary Figure 1 The cross-validation error rate for 577 eggplant samples with a K value of 2−10.

Supplementary Figure 2 LD decay plot of 577 eggplant samples.

Supplementary Figure 3 Histogram of the FSI frequency distribution of the F_2_ population.
