# Corrigendum: Development of a 50K SNP array for whole-genome analysis and its application in the genetic localization of eggplant (*Solanum melongena* L.) fruit shape

**DOI:** 10.3389/fpls.2025.1547317

**Published:** 2025-02-11

**Authors:** Chuying Yu, Qihong Yang, Weiliu Li, Yaqin Jiang, Guiyun Gan, Liangyu Cai, Xinchun Li, Zhiqiang Li, Wenjia Li, Min Zou, Yang Yang, Yikui Wang

**Affiliations:** ^1^ Vegetable Research Institute, Guangxi Academy of Agricultural Sciences, Nanning, China; ^2^ Vegetable and Flower Research Institute, Chongqing Academy of Agricultural Sciences, Chongqing, China

**Keywords:** eggplant, liquid-phase probes, fruit shape, BSA-seq, candidate gene

In the published article, there was an error in **Figure 4** as published. There was a mistake in order of the table when the material sources and phenotypes were entered, resulting in the material names and sources not matching the phenotypes. The corrected **Figure 4** and its caption appear below.

**Figure 4:**
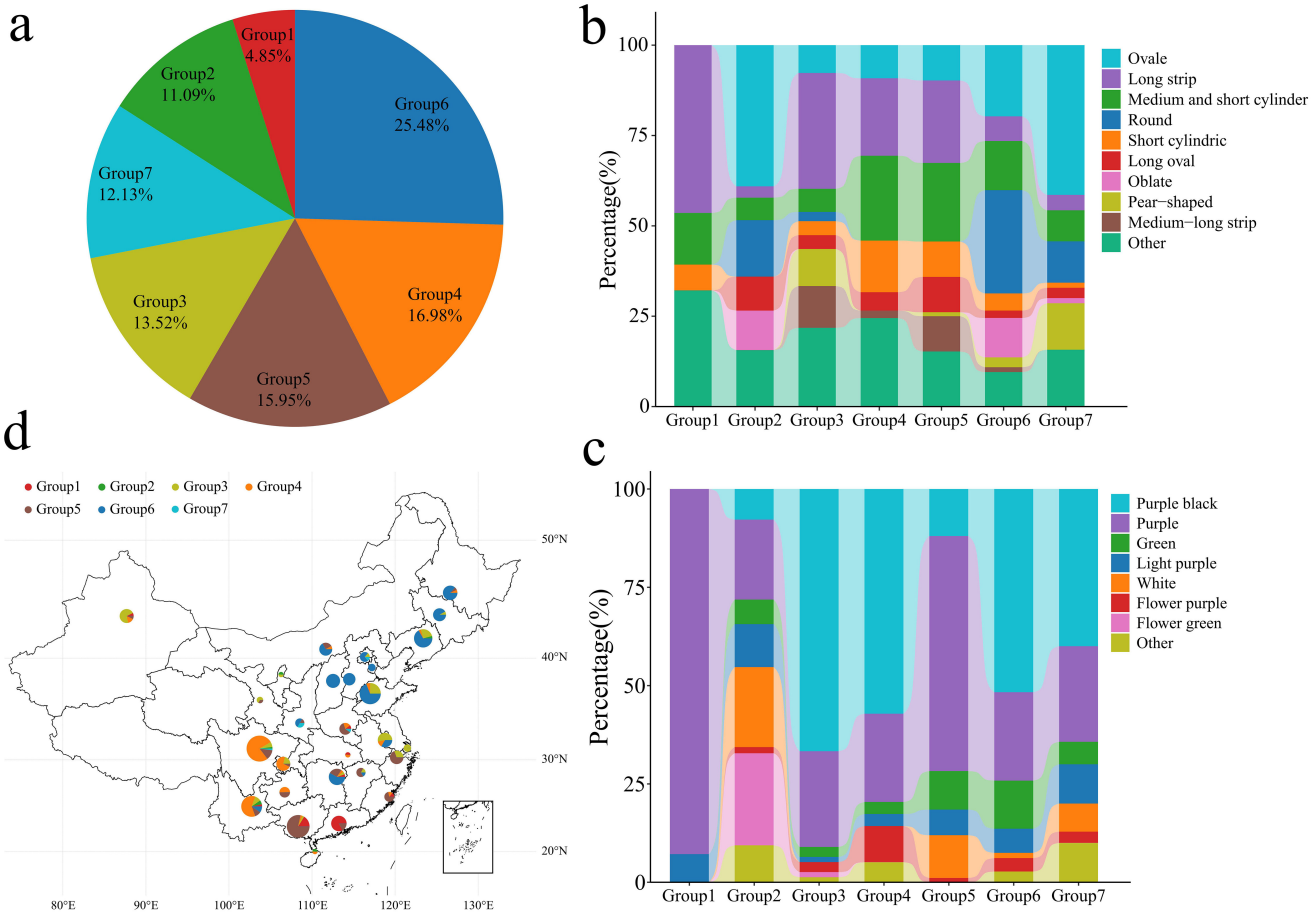
Classification, fruit shape and color analysis of 577 eggplant samples. **(A)** Proportion of 577 eggplant materials; different colors represent different groups. **(B)** Proportion of different eggplant fruit shapes in 577 groups. **(C)** The proportion of fruit colors in different groups of 577 eggplants. **(D)** Geographical distribution of Chinese eggplant materials; different colors represent different groups.

In the published article, there was an error in Supplementary Table S1. There was a mistake in the order of the table when the material sources and phenotypes were entered, resulting in the material names and sources not matching the phenotypes.

In the published article, there was an error in **Materials and methods**. This sentence previously stated:

“Of these, 428 were Chinese varieties (including 90 were purified offspring of self-selected varieties, and self-selected 15 eggplant lines), 149 were varieties from other countries (Supplementary Table S1)”

The corrected sentence appears below:

“Of these, 390 were Chinese varieties (including 78 were purified offspring of self-selected varieties, and self-selected 4 eggplant lines), 148 were varieties from other countries (Supplementary Table S1)”

In the published article, there was an error in **Results**. This sentence previously stated:

“Group 1 contained 33 genotypes, accounting for 5.72% of the total genotypes. Group 2 contained 49 genotypes, accounting for 8.49% of the total genotypes. Group 3 contained 96 genotypes, accounting for 16.64% of the total genotypes. Group 4 contained 117 genotypes, accounting for 20.28% of the total genotypes. Group 5 contained 107 genotypes, accounting for 18.57% of the total genotypes. Group 6 contained 129 genotypes, accounting for 22.36% of the total genotypes. Group 7 contained 46 genotypes, accounting for 7.79% of the total genotypes. Further analysis of the fruit shape of the 577 eggplants revealed that the fruits in Groups 1 and 4 were mainly long strips, medium−short tubes or short tubes (**Figure 4B**). The fruits in Groups 2 and 3 were mainly long strips, oval and medium−short tubes. The fruits in Groups 5 and 6 were mainly oval, round and flat round. The fruits in Group 7 were mainly short sticks, pears and long strips. Analysis of 577 eggplant fruits revealed that the fruits in Groups 1, 2, 3, 4, 5 and 7 consisted of mainly purple-black and purple-red. The fruits in Group 6 were mainly purple, white and purple-white (**Figure 4C**). Further analysis of the geographical distribution of the 428 Chinese eggplant materials revealed that Group 1 eggplant materials were distributed mainly in Sichuan and Yunnan, China (**Figure 4D**). Group 2 was mainly distributed in the northwest inland areas of China (Xinjiang, Sichuan, Shaanxi, Gansu and Guizhou). Group 3 was mainly distributed in the southern coastal areas of China (Yunnan, Guangxi, Guangdong, Zhejiang, Fujian and Jiangxi), with Guizhou and Heilongjiang containing 5 and 4 materials, respectively. Group 4 was distributed mainly in the central and eastern coastal areas of China (Shandong, Jiangsu, Shanxi, Chongqing, Inner Mongolia, Hebei, Liaoning, Anhui and Henan). Group 5 was mainly distributed in Northeast China (Liaoning, Jilin and Heilongjiang), with 8 materials in Beijing and 6 materials in Shandong and Tianjin. Group 6 consisted mainly of materials from other countries, with a small amount of materials in Yunnan, Guangxi and Hainan in China, and Group 7 did not contain Chinese materials]”

The corrected sentence appears below:

“Group 1 contained 28 genotypes, accounting for 4.85% of the total genotypes. Group 2 contained 64 genotypes, accounting for 11.09% of the total genotypes. Group 3 contained 78 genotypes, accounting for 13.52% of the total genotypes. Group 4 contained 98 genotypes, accounting for 16.98% of the total genotypes. Group 5 contained 92 genotypes, accounting for 15.95% of the total genotypes. Group 6 contained 147 genotypes, accounting for 25.48% of the total genotypes. Group 7 contained 70 genotypes, accounting for 12.13% of the total genotypes. Further analysis of the fruit shape of the 577 eggplants revealed that the fruits in Groups 1, 3, 4 and 5 were mainly long strips, medium−short tubes or short tubes (**Figure 4B**). The fruits in Groups 2, 6 and 7 were mainly oval, round and flat. Analysis of 577 eggplant fruits revealed that the fruits in Groups 1 and 2 consisted of mainly light purple and purple−red fruits. The fruits in Groups 3, 4, 5 and 6 were mainly purple−black and purple−red (**Figure 4C**). Further analysis of the geographical distribution of the 390 Chinese eggplant materials revealed that Group 1 eggplant materials were distributed mainly in Guangxi and Guangdong, China (**Figure 4D**). Groups 4 and 5 were distributed mainly in southern China (Yunnan, Guangxi, Chongqing, Guangdong, Zhejiang and Fujian). Group 6 was distributed mainly in the central and eastern coastal areas of China (Shandong, Liaoning, Shanxi, Heilongjiang, Hunan, Jilin, Hebei, Inner Mongolia, Jiangsu, Beijing, Tianjin, Yunnan, Shaanxi and Jiangxi). Groups 2 and 7 consisted mainly of materials from other countries, with small amounts of materials from Yunnan, Liaoning, Hainan, Ningxia, Beijing, Sichuan, Anhui and Shaanxi in China. An analysis of the phylogenetic tree, PCA, population structure and geographical distribution revealed that eggplants of the same subpopulation had a close geographical distribution.”

In the published article, there was an error in **Discussion**. This sentence previously stated:

“Groups 1, 2, 3, 4 and 5 were all Chinese eggplant materials, and Group 1 was located at the outermost branch of the evolutionary book, indicating that Sichuan and Yunnan in China are the origin centers of cultivated eggplant. Group 6 contained mainly materials from other countries, whereas Yunnan, Guangxi and Hainan in China contained a small amount of materials. Group 7 did not include Chinese materials.”

The corrected sentence appears below:

“Groups 4, 5 and 6 can distinguish materials from southern and northern China. Groups 2 and 7 consisted mainly of materials from other countries, whereas China contained a small amount of materials.”

The authors apologize for these errors and state that this does not change the scientific conclusions of the article in any way. The original article has been updated.

