# Supplementary figures and images for: Development of a 50K SNP array for whole-genome analysis and its application in the genetic localization of eggplant (Solanum melongena L.) fruit shape
Source: Front Plant Sci. 2024 Nov 25;15:1492242. doi: 10.3389/fpls.2024.1492242 (PMC11629150; doi:10.3389/fpls.2024.1492242)

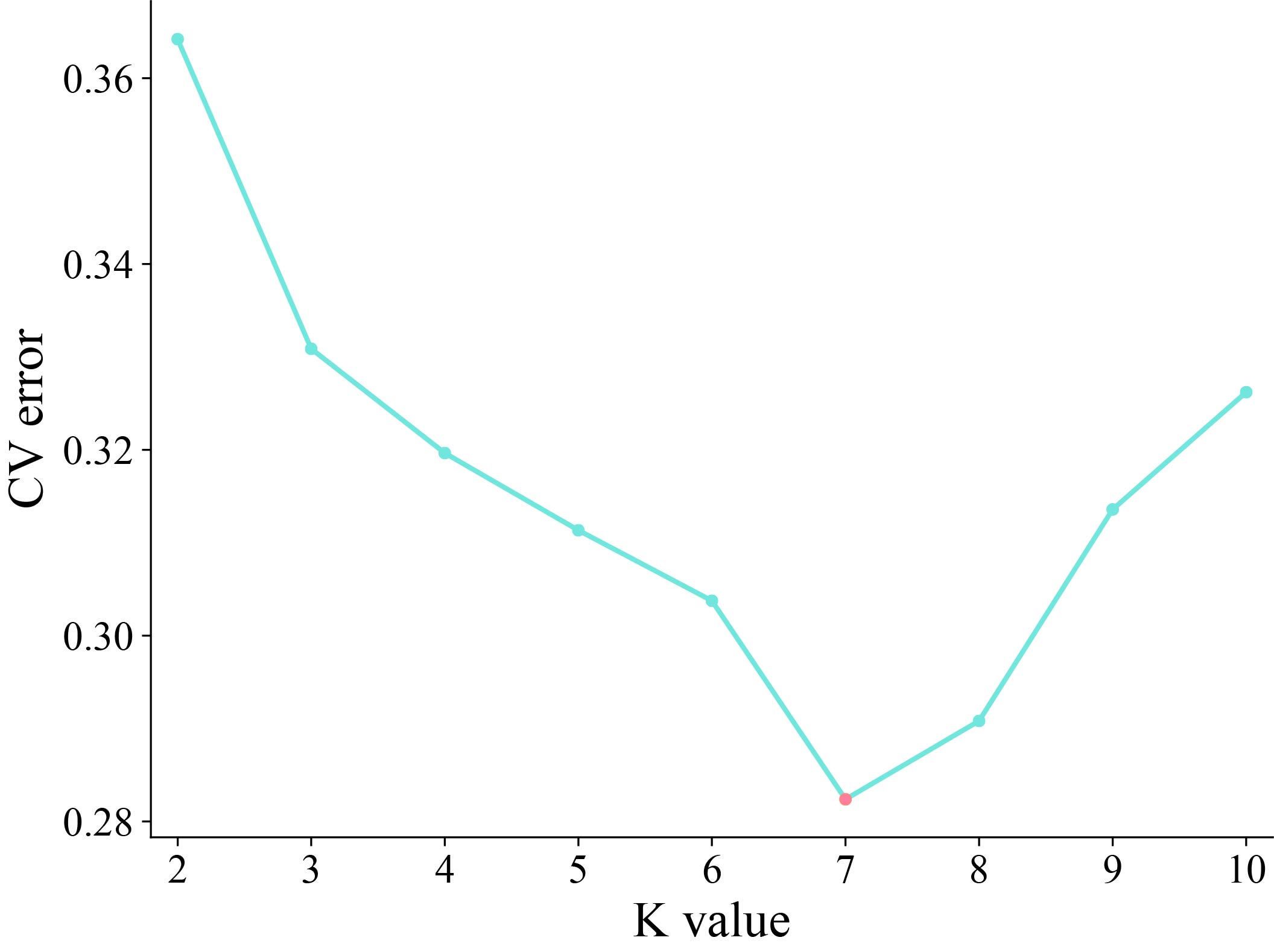

Supplement: Supplementary Figure 1 — The cross-validation error rate for 577 eggplant samples with a K value of 2−10. [file Image1.jpeg]

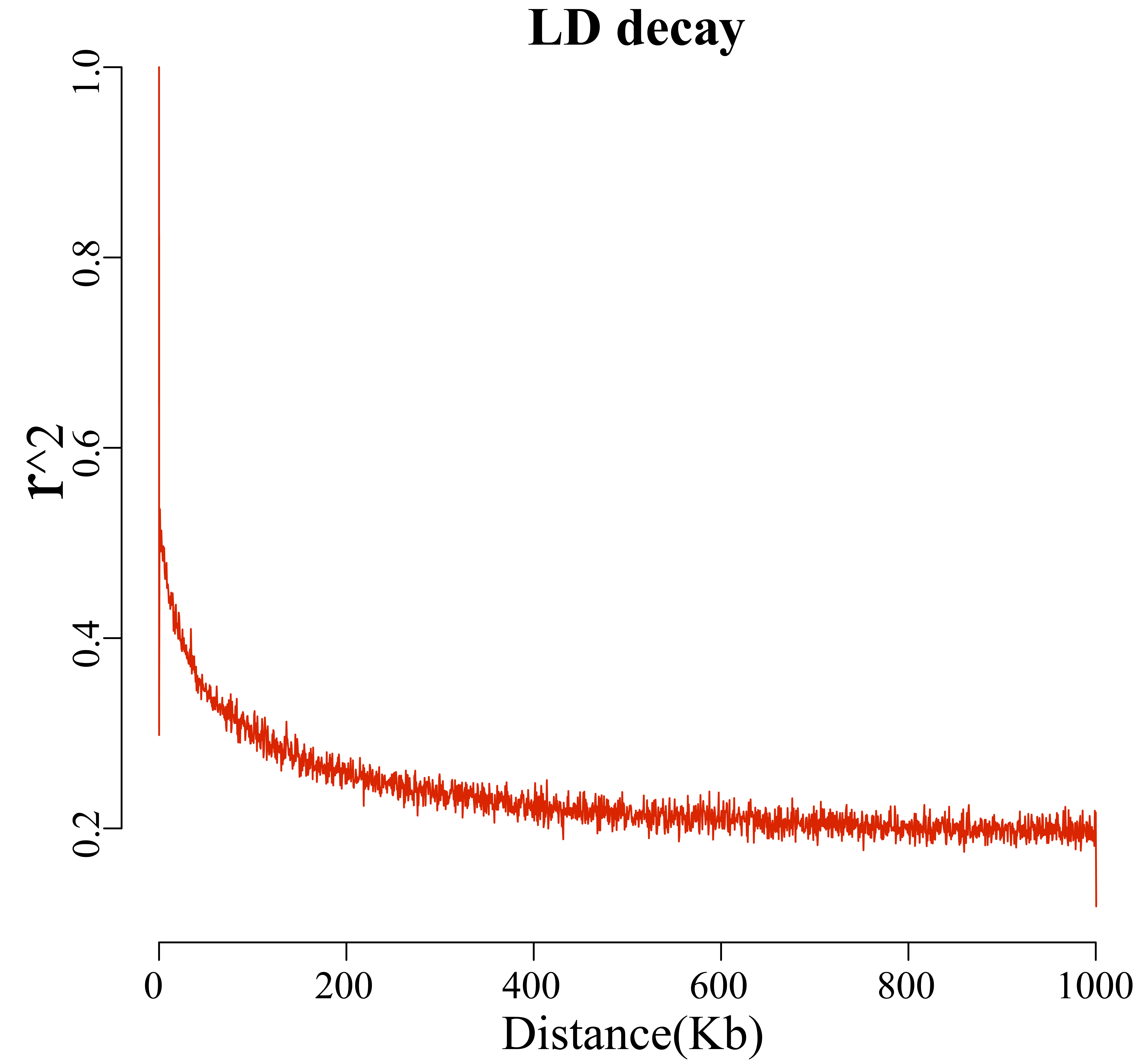

Supplement: Supplementary Figure 2 — LD decay plot of 577 eggplant samples. [file Image2.jpeg]

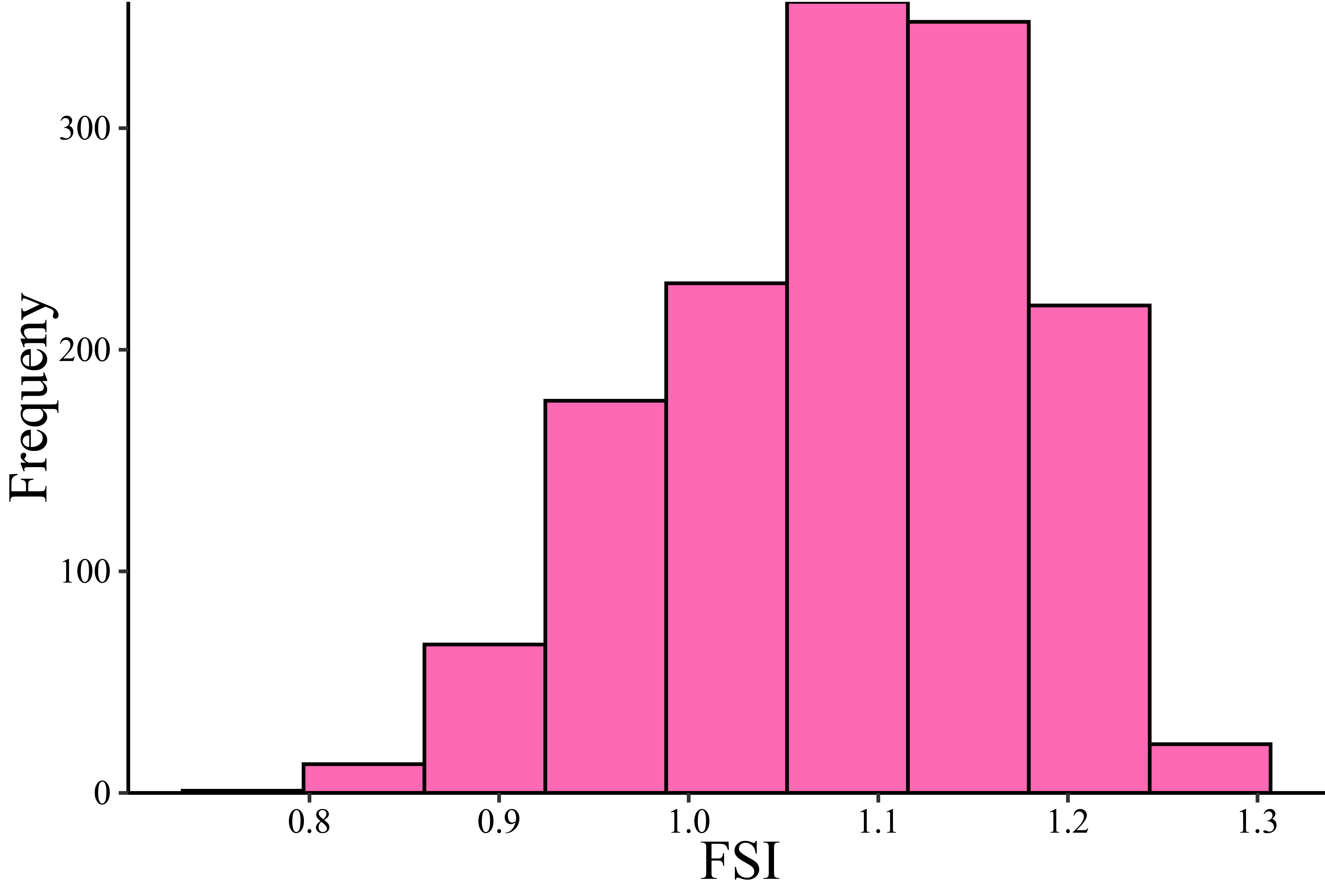

Supplement: Supplementary Figure 3 — Histogram of the FSI frequency distribution of the F2 population. [file Image3.jpeg]
